# The Effect of Fear of COVID-19 on Green Purchase Behavior in Pakistan: A Multi-Group Analysis Between Infected and Non-infected

**DOI:** 10.3389/fpsyg.2022.826870

**Published:** 2022-03-29

**Authors:** Kubra S. Sajid, Shahbaz Hussain, Rai I. Hussain, Bakhtawar Mustafa

**Affiliations:** ^1^Department of Management Sciences, University of Okara, Okara, Pakistan; ^2^The Evidence-Based Research Center for Educational Assessment, Jiangsu University, Zhenjiang, China; ^3^School of Business, George Mason University, Fairfax, VA, United States

**Keywords:** green purchase behavior, fear of COVID-19, mortality salience, psychological distress, COVID-19

## Abstract

The coronavirus disease 2019 (COVID-19) pandemic and its effects on an individual’s life have altered the consumer behavior. In the context of purchase and consumption, a shift from conventional to green purchase has been noticed. Although the factors underlying this shift were relatively unexplored, the study aimed to identify the factors that influenced a significant role in the green purchases during the outbreak and the relationship of these factors with green purchase behavior (GPB). Subsequently, this study investigates and interprets the role of fear of COVID-19 (FCV), psychological distress (PD), and mortality salience (MS) in predicting consumer’s GPB. This research adopted a quantitative methodology using data collected from 432 respondents in various cities across Pakistan. Smart-PLS 3 was used to evaluate the measurement model, structural model, and multi-group analysis (MGA). Despite having the negative psychological and physical impact of the pandemic, a significant proportion of customers have switched to healthier and sustainable products. This research revealed that the FCV, PD, and MS plays a substantial role in adopting GPB. All the direct relationships were positive and significant. In addition, MS and PD partially mediate the effect of FCV on GPB. Furthermore, the MGA revealed that the infected respondents were interested in purchasing green products than uninfected respondents due to their FCV; conversely, the PD and MS were higher in uninfected individuals than infected ones. However, there is a vast literature on GPB, but little has investigated the cumulative impact of FCV, PD, and MS on GPB.

## Introduction

In recent years, there has been a noticeable shift in the consumption pattern as it has made numerous changes and environmental damages, such as increased pollution, global warming, resource depletion, and reduction in flora and fauna, and these environmental changes also affect the health of individuals (Chen, 2010; Soomro and Mirani, 2020). As a result, the consumers have started to acknowledge that their purchase decisions and behaviors can have a detrimental effect on their health and the environment. Additionally, several organizations have begun to make organic and environment-friendly products, and a large group of customers is eager to embrace these products. Therefore, customers have shown an unprecedented interest in purchasing these products (Nimse et al., 2007). Environment-friendly, green, or sustainable products are those products that do not have any adverse effect on Earth, can be recycled, and are also beneficial for human health (Soomro and Mirani, 2020; Elsantil, 2021). In addition, for the improvement of society and environment, the green products have significantly contributed (Mala and Bencikova, 2018).

Green purchase behavior (GPB) refers to the purchasing of environment-friendly, sustainable, or green products. Several studies have been conducted on what motivates customers to purchase green products, and researchers have found that subjective norms (Witek and Ku, 2021), trust (Joshi and Rahman, 2015), environmental knowledge (Kaufmann et al., 2012), interpersonal influence (Cheah and Phau, 2011), media exposure (Lee, 2014), price (Soomro and Mirani, 2020), environmental concern (Wei et al., 2017), attitude and intention (Zheng et al., 2021), and demographic factors (Hojnik et al., 2019) significantly impact GPB. On the other hand, Pappas (2021) found that psychological factors substantially affect GPB, i.e., negative emotions can prompt eco-friendly, green, or sustainable behavior. Correspondingly, Muralidharan and Sheehan (2018) addressed the role of negative emotions in influencing GPB and found that women were more prone to green purchases than men because of green guilt. Similarly, fear can also motivate individuals to engage in preventive behavior (Green and Murphy, 2014). Moreover, scholars have found that fear has a substantial role in adhering healthy and decreasing unhealthy behavior (Bhuiyan et al., 2020; Lin, 2020). However, several studies have been conducted on GPB, but how the fear of coronavirus disease 2019 (COVID-19) (FCV) influences the GPB is limited. The COVID-19 is a contagious disease that causes severe acute respiratory syndrome coronavirus 2 (SARS-CoV-2). In Wuhan, China, the disease was discovered in December 2019 and has since spread globally, culminating in the ongoing coronavirus pandemic. Governments were paying attention and exerting effort to control the spread of diseases, i.e., strict lockdown, prohibition of outdoor activities, and wearing a mask. The first case in Pakistan was reported in February 2020, and by the end of 2020, the total number of confirmed cases was 482,178, with 10,176 deaths. Individuals became concerned about the COVID-19 because of its extremely high infection rate and the number of mortalities. It has been claimed that individuals were and still are afraid of contacting individuals who may have COVID-19 infections (Lin, 2020). The COVID-19 pandemic came not only with physical ailment but also with psychological, social, and economic outcomes globally. The “COVID-19” was the most-watched and rummaged topic on the internet and across all media platforms. Media platforms and internet played a vital role in spreading awareness about the COVID-19, but excessive information about the disease exacerbates psychological distress (PD) among the general public (Lorant et al., 2021). Additionally, the continuous increase in death tolls sends a reminder of mortality in individuals. Moreover, separation from family members, death of loved ones, getting infected, isolation, financial uncertainty, quarantine, school closure, and lockdown have a severe negative effect on mental health (Ambelu et al., 2021). In Beijing, China, the rate of depression and post-traumatic stress disorder (PTSD) in the general public varied from 4 to 7% after the COVID-19 pandemic (Mowbray, 2020). Therefore, the FCV induces psychological threats, i.e., MS and PD. The consciousness of inevitability and uncertainty of one’s death refers to MS (Becker, 1997), whereas PD refers to emotional suffering, i.e., depression and anxiety (Drapeau et al., 2012).

On the whole, the pandemic has affected every individual’s life, and individuals are now more apprehensive about their health. According to various studies, the pandemic has changed respondent’s perceptions and attitudes toward green consumptions because of its safer and healthier characteristics. Specifically, individuals were directed to be safe and healthy throughout the pandemic, safe in a way they should be at home to prevent themselves and others from getting infected. Similarly, consuming healthy food that would boost their immunity, make them strong was the concern of health departments. Undoubtedly, in the context of COVID-19, a more positive attitude toward the consumption of green products (i.e., organic food and eco-friendly) has been seen (Her, 2021). Since green purchases increase during the pandemic in Pakistan, it is essential to investigate factors that drive consumer’s GPB. As discussed above, negative emotions and fear have ability to affect GPB. The purpose of this study is to investigate the role of the FCV in green purchases, to examine the impact of FCV on PD and MS, and to investigate the effect of FCV on GPB *via* PD and MS. Lastly, the difference in the effect of FCV on PD, MS, and GPB between infected and uninfected individuals will be explored. This research is expected to provide more understanding of the connection between each variable and capture new insight to support further research related to the chosen variables and industry.

## Literature Review and Hypothesis Development

### Fear of COVID-19 and Green Purchase Behavior

The COVID-19 has created immense upheavals in our lives; fear and uncertainty during the pandemic inescapably impacted consumer behavior, indicating that consumers began to care more about their health and the environment. Furthermore, it has been seen that the fear generated by natural or non-natural calamities can encourage prosocial behavior (Sun et al., 2021) and also the COVID-19 pandemic has changed consumer behavior in more sustainable and healthier directions (Ben Hassen et al., 2020). According to Webmd (2020), the pandemic has caused us to reconsider how to maintain the environment, which will protect the health of others. In 2020, according to a poll created by the Association of Manufacturers and Distributors, 44% of consumers had stopped buying items from brands that they did not perceive as sustainable (AECOC, 2020). Notably, individuals were very concerned about the environment and their health, and they were willing to pay for green products though they have high prices. Similarly, the effect of the COVID-19 pandemic on the purchase of the green products was also significantly affected (Vaishali and Hemanathan, 2020), because the pandemic encouraged the individuals to be in healthy behavior, whether related to the environment or their health. According to the results of Qi et al. (2020) study, the COVID-19 crisis impacted green purchases and increased green product purchase intention among Chinese consumers. In addition, it has been found that COVID-19 changes the perception of green consumption, organic food, and food safety among Malaysians (Safuan et al., 2021). Furthermore, in the context of COVID-19 pandemic, a focus on eating healthier diet and environmental awareness has been prominent and individuals were worried about the environment and its effect on their health (Naja and Hamadeh, 2020; Ali Q. et al., 2021). Evidently, the coronavirus pandemic has affected individual’s feelings and thoughts, hygiene practices, and consumption patterns. As a result of the pandemic, there has been a rise in the consumption of green and organic foods. On the basis of this, we develop the following hypothesis,

H1: Fear of COVID-19 positively and significantly influences GPB

### Fear of COVID-19 and Psychological Distress

During the pandemic, several variables may lead to mental health deterioration and increased suffering, such as fear of getting infected and worries about the health of others, financial uncertainty, job and school closures, and lessened social contacts (Brooks et al., 2020; Shevlin et al., 2020). The unpleasant and traumatic events, such as the present epidemic or other calamities, ends up causing apprehension and, in some circumstances, obsessive thoughts that are unsolicited and recurring, producing misery, and are also linked to other stress-related diseases, i.e., depression, distress, death anxiety, obsessive disorder, and other psychological disorders (Marroquín et al., 2020). Some early studies in China revealed that 25% of general public had experienced anxiety and PD in response to the outbreak of COVID-19 (Qiu et al., 2020; Wang et al., 2020). Evidently, the COVID-19 related information kept individuals updated on how to stay safe, but becoming overly concerned with the disease has unhealthy repercussions (Bakshi et al., 2021). Moreover, anxiety and fear-related distress are also caused by the fear of getting infected or contaminated (Taylor et al., 2020). Therefore, FCV was considered the leading cause of PD among the general public (Di et al., 2021; Lorant et al., 2021). As the number of cases grew, governments worldwide began sealing borders, instituting social distancing restrictions, and lockdown orders to slow down the virus spread. However, all these restrictions slowed down the propagation of the virus, but it created a fuss among the public. Several studies have found that the fear of getting infected causes PD around the world (Brooks et al., 2020; Moh et al., 2021). Fenollar-Cortés et al. (2021), explicated that during quarantine, women showed more negative emotions, such as PD than men. Furthermore, survey conducted by Daly and Robinson (2021) concluded that there is a sharp rise in distress levels during the pandemic in the United States and Rillera et al. (2021) found that the PD levels vary across different countries, the respondents in Sri Lanka hold the lowest level of PD than Nepal but the respondents from Vietnam holds the highest level of PD because of FCV. In Pakistan, limited studies have been conducted to evaluate the PD level because of FCV. Based on this, we develop the following hypothesis,

H2: Fear of COVID-19 positively and significantly influences PD

### Fear of COVID-19 and Mortality Salience

During the pandemic, the death rate of infected patients was very protuberant, which induced death reminders among individuals. Subsequently, the MS triggers anxiety about one’s death and causes colossal concern about the death of infected loved ones (Cable and Gino, 2020; Evers et al., 2021). According to Ahorsu et al. (2020), the item “I am afraid of losing my life because of COVID-19 virus” has the highest factor loading on the FCV Scale which implies that individual’s concern about mortality threat was strongly predictive of widespread fear about the virus. Moreover, the fear of getting infected and death was found to be greater in young women than men (Quadros et al., 2021), and a study found that men were more scared of getting infected (Mertens et al., 2020). Evidently, the most severe psychological threats related to COVID-19 were fear and anxiety of death (Courtney et al., 2020), caused by the deadly reminder of the rise in fatalities and infected patients. As the death toll increased, not only infected patients but also the uninfected were afraid to succumb to the COVID-19 and lose their lives. Thus, the FCV causes MS (Hu et al., 2020) and MS was found higher in elders than young ones during the pandemic (Guner et al., 2021). A study conducted by Newton-John et al. (2020) investigated the anxiety of death because of the virus among Australian respondents and found that 22% of respondents were afraid of dying because of COVID-19. In Pakistan, the death anxiety because of MS was higher in non-working than working individuals (Shakil et al., 2021). Hence, FCV induced MS which further causes death anxiety among the public around the globe. On the basis of this, we formulated the following hypothesis,

H3: Fear of COVID-19 positively and significantly influences MS

### Psychological Distress and Green Purchase Behavior

Pandemics and natural disasters are highly stressful that can easily trigger negative emotions and mental health problems. Furthermore, a link was found between psychological factors (anxiety, stress, depression, fear, and so on) and consumer behavior; studies have found that these factors have both negative and positive effects on purchase behavior depending on the product categories (Di Crosta et al., 2021). A condition of emotional discomfort, also associated with anxiety and depression symptoms, is defined as PD (Mirowsky and Ross, 2002). The COVID-19 came up with lots of negative impacts on an individual’s mental health, and it has also been seen that the negative impacts sometimes lead to positive outcomes. Researchers have found that PD significantly impacts an individual’s health, and sometimes they find coping strategies to reduce the distress. A study conducted by Ashraf et al. (2021) found that searching for meaning in life and feeling satisfied may reduce PD.

Additionally, the purchase of green, sustainable, organic, or eco-friendly products during pandemics has increased owing to their distinguished physical as well as psychological benefits. Similarly, studies have shown that the customers engaged in GPB got psychological benefits (Kushwah et al., 2019). Consuming green products made customers healthy, safe, and comfortable, and the psychological effects leading to consumer’s preference for green purchases, i.e., organic food (González et al., 2019; Boobalan et al., 2021). Several studies have been conducted on the impact of psychological factors on purchase behavior (Rathod and Bhatt, 2013; Durmaz, 2014), but the effect of PD related to FCV on GPB is still unexplored. On the basis of this we develop the following hypothesis,

H4: Psychological distress positively and significantly influences GPB

### Mortality Salience and Green Purchase Behavior

Disasters entail not just physical but also psychological threats, such as the fear of death (Linley and Joseph, 2006). Similarly, the COVID-19 has evolved into a continuous threat to life, raising mortality awareness. MS, awareness of death that produces death anxiety among individuals (Becker, 1997), whereas GPB (GPB) refers to the purchase of green, sustainable, or eco-friendly products. According to the literature when individuals are being exposed to the threats, then they will show positive behavior, willingness to contribute in the charitable organization (Jonas et al., 2002; Ferraro et al., 2005), sports and fitness activity (Arndt et al., 2003), purchase of organic food (Mandel and Smeesters, 2008), home-made products (Nelson et al., 1997) and so on. Therefore, it has been found that attitude and behavior are substantially influenced by MS (Steele, 1975). Another role of MS is that it has ability to increase the purchase and consumption of sustainable products (Mandel and Smeesters, 2008). Furthermore, the fear and thought of death have ability to motivate individuals to engage in healthy behavior, as unhealthy behaviors affect their physical and mental health (Pyszczynski et al., 2020). Moreover, Zaleskiewicz et al. (2015) conducted a study and they found that MS increases the desire for green purchases because consuming green products help to achieve a sustainable environment and benefits individual’s health. Again, when MS is high among individuals, their concern for their health and environment will be increased, and this health and environmental concern affect their purchase decisions (Nekmahmud and Fekete-Farkas, 2020). Indeed, the reminders of death can potentially affect customer decisions in ways that they are not even aware of it. Despite the scarcity of literature on MS and GPB, certain studies show an influence of MS on GPB (Rahimah et al., 2020). On the basis of this, we develop the following hypothesis,

H5: Mortality salience positively and significantly influences GPB

### Mediating Role of Psychological Distress and Mortality Salience

During the COVID-19 outbreak, the public was continually watching the news related to COVID-19 to stay informed, but the rise in the number of infections and deaths instilled terror. So, the individuals became very concerned about the COVID-19 because of its exceptionally high infection, fatality rate, and its pandemic nature exacerbate fear worldwide. The FCV is directly associated with the psychological outcomes, i.e., hopelessness, anger, anxiety, PD, MS, and the fear of getting infected found in the individuals who are infected and uninfected (Rogers et al., 2020; Rubin and Wessely, 2020). Additionally, the pandemic has increased death anxiety by raising the awareness of vulnerability among individuals, and the psychological comfort was also affected by the COVID-19 related information. Therefore, FCV caused PD and MS in public, and to alleviate this menacing effect, individuals find coping strategies (Pyszczynski et al., 2020). Additionally, it has been seen that death threats considerably enhanced individual’s consumption behavior (Song et al., 2020) and the mortality reminder differentially affects health behavior and also slows down the spread of the virus (Courtney et al., 2021). Surprisingly, consuming healthy, eco-friendly, and sustainable products reduces PD (Kushwah et al., 2019). In addition to this, the studies have found that PD and MS increase the effect of FCV (Menzies and Menzies, 2020; Ashraf et al., 2021), and they impact the purchase behavior of consumers. Still, fewer have investigated the mediation of MS and PD between the FCV and GPB. So, we formulated the following hypotheses,

H6: Psychological distress mediates the effect of FCV on GPB

H7: Mortality salience mediates the effect of FCV on GPB

Moreover, the PD because of FCV was found more in infected than uninfected individuals (Feng et al., 2020). Similarly, Mohammadian Khonsari et al. (2021) conducted a study on infected and uninfected healthcare workers and found that infected workers showed more psychological symptoms, i.e., death anxiety, distress, and depression, than uninfected workers whereas Xiao et al. (2020) found that the fear of getting infected and threat to life was higher in uninfected individuals. As a result, more research is required to know the difference in the impact of FCV on PD, MS, and GPB between infected and uninfected people. Based on this, we formulated the following hypothesis,

H8: The impact of FCV on PD, MS, and GPB differs in infected and uninfected group

### Green Purchase Behavior

According to the protection motivation theory (PMT), the fear factors significantly impact health habits and behaviors; if the fear is persistent, individuals will avoid or escape it (Rogers, 1975). The PMT includes two cognitive processes, hazard identification and coping assessment. According to Floyd et al. (2000) threat appraisal and coping assessment generate understanding, which improves an individual’s ability to respond to a peril. Moreover, people will defend themselves and others if threat evaluation and coping assessment are appropriately managed. Furthermore, any intimidating situation, such as a pandemic or heath catastrophe push individuals to involve in behaviors that are essential for existence. Additionally, the factors that root behavioral changes are fear and anxiety, instigated by insecurity and instability. A previous research has revealed that external events that threaten an individual’s wellbeing stimulate restorative reaction processes to alleviate fear and anxiety (Arndt et al., 2004; Maheswaran and Agrawal, 2004). These reaction processes can cause individuals to purchase to attain a sense of stability, comfort, and a temporary escape, which can also function as a reimbursing method to relieve stress.

Green purchase behavior is buying environment-friendly, reusable, or disposable items and avoiding products that are hazardous to the environment and health (Chan, 2001; Mostafa, 2007). Moreover, green products are organic products (dairy, vegetables, fruits, skin, and beauty products, poultry, etc.) that are beneficial for health and the environment. Consuming organic food benefits not only consumer’s health but also encourages agriculturalists and manufacturers to avoid using excessive chemicals, which may increase the output but harm the environment. Currently, consumers are considering the influence of the environment and believe that green products will provide future benefits to the environment and human health; such advantages compel them to take significant moral duties by preferring green over non-green products. However, there have been a lot of research on GPB, (Ru et al., 2018; Gao et al., 2020), organic food (Singh and Verma, 2017; Latiff et al., 2019), and green consumption (Joshi and Rahman, 2015); it has been considered that the green products are purchased by the individuals who have great knowledge about environmental and health issues, and they know how to solve these issues (Chan and Lau, 2000; Albayrak et al., 2013). During the pandemic, severe health and environmental challenges were observed, as well as a significant shift toward green products. The purpose of this study is to elucidate what induces people to buy green products during the pandemic.

**FIGURE 1:**
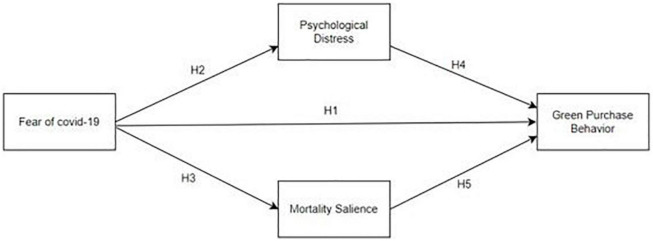
Conceptual model.

The proposed model is outlined in Figure 1.

## Materials and Methods

Consumers who were involved in the green products purchases were the population studied in this study. A convenience sample of 432 consumers was drawn from various cities across Pakistan using the non-probability sampling technique because the time was limited, and through this technique, sufficient reliable data can be collected in a very short period of time. For the analysis, quantitative data collected *via* an online survey (Questionnaire) was used. The questionnaire was adapted in English to ensure that the questions were easily understood by the respondents. The first section of the questionnaire contains demographic profile of respondents, i.e., gender, age, employment status, income, and COVID-19 infection (infected; who are still infected with COVID-19. Uninfected; individuals who are not infected). The demographic information is presented in Table 1. The second section contains the items of variables. Individual responses were analyzed using a “Seven-point Likert Scale.” The scale was extended from 1 to 7, with 1 indicating strongly disagree and 7 indicating strongly agree. The scales used in the study to measure the variables are shown in Table 2. In assessing reliability and validity (i.e., goodness of measures), testing the structural model, and assessing differences in infected and uninfected groups, we used partial least squares structural equation modeling (PLS-SEM) version 3.0.

**TABLE 1:** Demographic profile.

Characteristics	Frequency	%
**Gender**		
Female	249	57.7
Male	183	42.3
**Age**		
Below 20	35	8.3
20–29	248	57.6
30–39	114	26.4
40–49 and above	35	8.3
**Employment**		
Employed full-time	174	39.6
Employed part-time	86	20.1
Unemployed	172	40
**Monthly income**		
Less than Rs.25,000	38	9
Rs25,000–50,000	101	23.6
Rs50,000–100,000	80	18
Rs10,000–150,000	75	17.4
Rs150,000–200,000	138	32
**COVID-19 infection**		
Infected	172	39.8
Uninfected	260	60.2

**TABLE 2:** Measures and their sources.

Measures	Items	Source
Green purchase behavior	7 items	Booi Chen and Teck Chai, 2010
Fear of COVID-19	7 items	Ahorsu et al., 2020
Psychological distress	14 items	Feng et al., 2020
Mortality salience	15 items	Templer, 1970

## Results

### Measurement Model

The reflective constructs of the proposed model are evaluated using reliability and validity tests. Several criteria are examined to assess validity and reliability, such as outer loadings, average variance extracted (AVE), discriminant validity, composite reliability, and Cronbach’s α value. Table 3 shows that no measurement items were removed because the outer loadings have high values. According to Hair et al. (2017), convergent validity will be established if AVE, composite reliability, and outer loadings are greater than 0.50, 0.70, and 0.60, respectively. Table 3 shows all the values are within the prescribed range.

**TABLE 3:** Items, Cronbach’s α, standard loading, composite reliability, and average variance extracted (AVE).

Constructs	Items		Standardized loading	Cronbach’s alpha	CR	AVE
**Fear of COVID-19**				0.914	0.932	0.662
	FCV-1	I am most afraid of coronavirus-19.	0.802			
	FCV-2	It makes me uncomfortable to think about coronavirus-19.	0.751			
	FCV-3	My hands become clammy when I think about coronavirus-19.	0.87			
	FCV-4	I am afraid of losing my life because of coronavirus-19.	0.826			
	FCV-5	When watching news and stories about coronavirus-19 on social media, I become nervous or anxious.	0.73			
	FCV-6	I cannot sleep because I’m worrying about getting coronavirus-19.	0.852			
	FCV-7	My heart races or palpitates when I think about getting coronavirus-19.	0.854			
**Mortality salience**				0.928	0.942	0.699
	MS-1	When it comes to COVID-19, I am very much afraid to die	0.823			
	MS-2	When it comes to COVID-19, the thought of death often hits my mind	0.831			
	MS-3	I feel nervous when thinking of the death scene caused by COVID-19	0.787			
	MS-4	I feel terrified if I got quarantined and medical treatment because of COVID-19 infection	0.777			
	MS-5	I am scared of COVID-19 infection	0.839			
	MS-6	I am terrified of death caused by COVID-19	0.841			
	MS-7	When it comes to COVID-19, The thought of death never bothers me.	0.828			
	MS-8	When it comes to COVID-19, I constantly feel that time flies and time is short	0.852			
	MS-9	When it comes to COVID-19, I fear dying a painful death	0.824			
	MS-10	The topic of COVID-19 death makes me feel worried and resignation	0.862			
	MS-11	I was afraid I do not have enough immunity to resist the Coronavirus effectively	0.835			
	MS-12	When it comes to COVID-19, I often think of how fragile life is	0.791			
	MS-13	I shudder at the thought of the heavy casualties of the COVID-19	0.837			
	MS-14	I feel scared and sad when I saw the news about the numbers of deaths caused by the COVID-19	0.7			
	MS-15	When it comes to COVID-19, I’m worried about the future	0.676			
**Psychological distress**				0.962	0.965	0.654
	PD-1	If I was infected with COVID-19, I might not be able to recover from it.	0.796			
	PD-2	When talking to a stranger, I would suspect that s/he might be infected with COVID-19.	0.814			
	PD-3	I am afraid to travel to places hard-hit by COVID-19.	0.792			
	PD-4	When I see an increase in the number of COVID-19 patients on the news, I feel anxious.	0.801			
	PD-5	When I see someone sneeze, I suspect s/he might be infected with COVID-19.	0.838			
	PD-6	I think frequent hospital visits would make it easier to be infected with COVID19.	0.842			
	PD-7	I fear to see the doctors and nurses who had worked in COVID-19 isolation wards.	0.826			
	PD-8	I think frequent use of air, train, bus and other public transport would make it easier to be infected with COVID-19.	0.86			
	PD-9	When I notice someone running a fever, I suspect s/he might be infected with COVID-19.	0.875			
	PD-10	When I see someone vomiting, I suspect s/he might be infected with COVID-19.	0.789			
	PD-11	I fear to live nearby a COVID-19 isolation hospital.	0.816			
	PD-12	When I see someone coughing, I suspect s/he might be infected with COVID-19.	0.872			
	PD-13	When I see someone without a mask, I suspect s/he might be infected with COVID-19.	0.759			
	PD-14	I suspect there were novel coronavirus in the air when there were people around.	0.816			
**Green purchase behavior**				0.963	0.966	0.675
	GPB-1	I often buy organic products.	0.775			
	GPB-2	I often buy products that are labeled as environmentally safe.	0.886			
	GPB-3	I often buy products that are against animal-testing	0.848			
	GPB-4	I often buy products that contain no or fewer chemical ingredients	0.824			
	GPB-5	When I consider buying a product, I will look for a certified environmentally safe or organic stamp.	0.887			
	GPB-6	I often buy products that support fair community trades.	0.872			
	GPB-7	I often buy products that use recycled/recyclable packaging.	0.749			

*AVE (Average Variance Extracted), CR (Composite Reliability).*

The discriminant validity is assessed using the Fornell–Larcker and Heterotrait–Monotrait ratio (HTMT) criteria. In Fornell–Larcker criterion, the off-diagonal values for each construct must be less than the square roots of AVE values. The discriminant validity was determined by comparing the square root of each AVE in the diagonal with the correlation coefficients (off-diagonal) for each construct in the relevant rows and columns, the square root of each construct’s AVE is higher than its correlation with another construct. This criterion is also met as shown in Table 4 (Fornell and Larcker, 1981).

**TABLE 4:** Discriminant validity—Fornell–Larcker criterion.

Constructs	FCV	GPB	MS	PD
FCV	*0.813*			
GPB	0.501	*0.836*		
MS	0.609	0.543	*0.808*	
PD	0.613	0.577	0.800	*0.821*

*Diagonal (italic) values are the square root of the AVE values of each respective construct.*

Additionally, all the values must be less than 0.90 to meet the HTMT criterion (Henseler et al., 2015; Hair et al., 2017). The HTMT ratio is measure of similarity between variables. If the values are smaller than 0.90 than the discriminant validity is established. If the values are greater than 0.90 than there is lack of discriminant validity. The value less than 0.90 depicts that the latent variables are reliably distinguishable. The HTMT ratio is less than 0.90 among all constructs: this criterion is also met as shown in Table 5. As a result, the discriminant validity of the constructs has been established. The structural model will be examined after the measurement model has been verified as valid and reliable.

**TABLE 5:** Discriminant validity—Heterotait-Monotrait (HTMT) ratio.

Sr. no	Constructs	HTMT correlations			
		1	2	3	4
1	Fear of covid-19				
2	Green purchase behavior	0.539			
3	Mortality salience	0.638	0.571		
4	Psychological distress	0.637	0.592	0.831	_

*The criterion for HTMT ratio is below 0.90.*

### Structural Model

**FIGURE 2:**
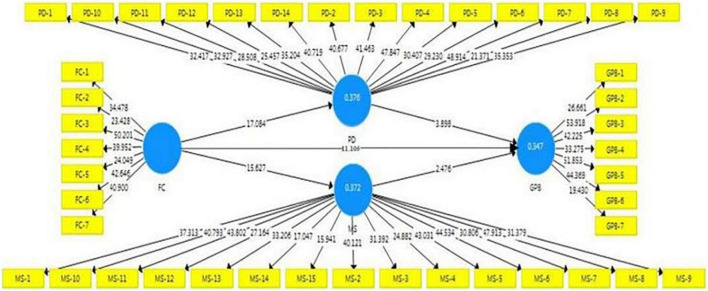
The bootstrapping result.

In the Smart PLS-SEM 3.0, a non-parametric procedure known as bootstrapping is used to test the model’s significance. In the bootstrapping method, predictive accuracy, predictive relevance, and path modeling are all calculated. The *R*^2^-value (0.347) and the results of blindfolding algorithm shows high *Q*^2^ (0.235) effect size for GPB. Furthermore, the bootstrapping procedure calculates the standardized root mean square (SRMR) value to assess the model fitness. For the structural model used in this study, the SRMR value is 0.064, which is within the acceptable range of 0–1 (Hooper et al., 2008). Figure 2 depicts the model derived from the bootstrapping procedure. After evaluating hypotheses, we have found that FCV, PD, and MS positive and significant influence on GPB, FCV positive and significant influence on PD and MS. Consequently, all hypotheses H1, H2, H3, H4, and H5 are accepted. The results are presented in Table 6.

**TABLE 6:** Structural relationship and hypothesis testing.

Hypothesis	Path	Path coefficient	T statistics	*P*-values	Decision
H1	FCV -> GPB	0.503	11.105	0	Supported
H2	FCV -> PD	0.613	17.084	0	Supported
H3	FCV -> MS	0.61	15.627	0	Supported
H4	PD -> GPB	0.381	3.898	0	Supported
H5	MS -> GPB	0.238	2.476	0.014	Supported

*p < 0.05.*

### Mediation Analysis

The results reveal that FCV has strong direct effect on GPB. On the other hand, the indirect impact of FCV on GPB through MS and PD is also significant. Consequently, the direct and the indirect impacts are significant. We also computed the variance accounted for (VAF), which determines the intensity of the indirect effect to the total effect (VAF = indirect effect/total effect), obtaining values of 45.61% (MS) and 51.59% (PD) between FCV and GPB. As a result, MS and PD partially mediate the relationship between FCV and GPB. Hypotheses H6 and H7 are thus accepted. Table 7 displays the results.

**TABLE 7:** Mediation analysis.

Path	Indirect effect	Total effect	VAF (indirect effect/total effect)	Type of mediation
FCV -> MS -> GPB	0.229	0.502	0.4561 = 45.61%	Partial
FCV -> PD -> GPB	0.259	0.502	0.5159 = 51.59%	Partial

*VAF, Variance Accounted for.*

### PLS-Multi-Group Analysis

In PLS-multi-group analysis (PLS-MGA), there is a significant difference between comparing groups if the values of *p* are greater than 0.95 and less than 0.05 (Sarstedt et al., 2011; Henseler et al., 2016). Finally, we analyze whether there were any differences in the effect of FCV on PD, MS, and GPB between the different groups studied in the paper (i.e., infected and uninfected individuals). MGA was used to test hypothesis H8 (Table 8). The values of *p* in PLS-MGA were less than 0.05, indicating that the impact of FCV on PD, MS, and green purchase behavior differs in the infected and uninfected group. As a result, our findings support hypothesis H8.

**TABLE 8:** Multi-group analysis.

Path	Path coefficients-diff (infected—uninfected)	*P*-value (infected vs. uninfected)
FCV -> GPB	*0.227*	*0.033*
FCV -> MS	*–0.165*	*0.038*
FCV -> PD	*–0.212*	*0.003*
MS -> GPB	0.149	0.420
PD -> GPB	–0.343	0.053

*The italic values show the significant difference.*

## Discussion

The COVID-19 pandemic has caused changes in human behavior, such as changes in consumption patterns and behaviors: it has also altered lifestyle, purchase intention, needs and wants, and the ways goods and services are consumed. During the pandemic, the one notable trend was an increase in green purchases. However, several studies have been conducted on GPB, but the factors that influence the COVID-19 pandemic were still unexplored. This research addressed this gap and found that GPB is influenced by the FCV, MS, PD. Additionally, the *R*^2^ value confirms that FCV, PD, and MS are good predictors of GPB. In the literature, GPB was influenced by negative emotions (Muralidharan and Sheehan, 2018). The standardized path coefficient FCV-GPB (0.503) in the structural equation model indicates that the FCV positively and significantly influences GPB. The value shows that because of the FCV, the consumer behavior evolves toward healthier and sustainable patterns. The effect of fear is based on the cognitive process, which sways individual’s attitudes and emboldens behavioral changes. Evidently, during the COVID-19 pandemic, a significant proportion of consumers have shifted to sustainable and healthier buying behavior (Borsellino et al., 2020; Orîndaru et al., 2021). The findings are not surprising, as if consumers are more concerned about their health and environment, they will be buy green products (i.e., organic food and eco-friendly products), and it is confirmed that the green products are their first choice, as they are conscious about their health and the environment. Furthermore, the individuals took extra precautions to be in more sustainable conduct, such as wearing masks, using sanitizers, social distancing to avoid getting infected, and eating healthier food to boost their immunity. So, the reason for the rise in green products purchases was FCV.

The results are consistent with the literature, which suggested that when the FCV is high in individuals, they experience an increased level of death anxiety and PD (Ho et al., 2020; Curşeu et al., 2021). Furthermore, the path coefficients FCV-MS (0.610) and FCV-PD (0.613) indicate that MS and PD are significantly influenced by FCV. The values indicate that FCV increased the MS and PD in the consumers during the pandemic. As the news related to COVID-19 infection, mortality, travel restrictions, isolation, and the lockdown has made everyone aware of the situation; it also instills fear among the public. Individuals were worried about their own and their loved one’s death, which also had negative effect on the psychological state (Moh et al., 2021; Rillera et al., 2021). Hence, FCV reminds that death can also cause psychological discomfort and that’s why the pandemic was critically associated with mental illness. On the other hand, the path coefficients MS-GPB (0.238) and PD-GPB (0.381) show that the MS and PD influence the GPB positively and significantly, and GPB is more influenced by PD than MS. According to the literature, when the MS is high, consumers would reassess their purchase decisions and gravitate toward products that benefits them while also lessening their anxiety about death (Rahimah and Khalil, 2018; Rahimah et al., 2020). The finding is aligning with the literature, as the MS was high during pandemic, consumers were searching for those products that would benefit them and the environment (Cox et al., 2021). Similarly, the PD during the pandemic was high among public (Daly and Robinson, 2021; Shakil et al., 2021), the high stress levels not only affect health but also decision making; consumers want to purchase products that benefit them psychologically, and buying green products reduces their stress level because of their benefits to the health and the environment (Boobalan and Sulur, 2020; Boobalan et al., 2021). This implies that the PD is more impactful than MS in purchasing green products (i.e., organic food). Hence, the findings support the literature that PD and MS substantially effect the green purchasing behavior. The FCV has positively impact MS and PD. Furthermore, FCV positively and significantly impact GPB through PD and MS, in the presence of MS and PD. Thus, MS and PD partially mediates the effect of FCV on GPB. Hence, the FCV directly and indirectly affects GPB.

In this study, MGA was also conducted between infected and uninfected groups. Significant differences are found in the impact of FCV on MS, PD, and GPB. The path coefficient of FCV-GPB (*p* of 0.033) shows a considerable difference and the values higher in infected than uninfected respondents, implying that the individuals infected with the COVID-19 have more FCV and are more enthused about acquiring green products than uninfected ones. As mentioned above, green or organic products significantly impact health; infected patients are more concerned about getting recovered soon, and after recovery, they do not want to give the virus a second chance, so they adopt healthy habits to stay well and resilient. As a result of their concern about becoming healthy and never getting infected again, they prefer green products (i.e., organic food). The path coefficient FCV-MS (*p* of 0.038) shows the substantial difference, and the values are higher in uninfected respondents than infected ones, indicating that uninfected individuals have a higher level of MS because of FCV than infected ones. This implies that the fear of death is more in uninfected people because they were kept up to date on the mortality in the past and which is still causing death anxiety among them. Furthermore, seeing the entire world die due to a horrendous epidemic makes people more concerned about themselves and their loved ones. Similarly, the path coefficient FCV-PD (*p* of 0.003) suggests that PD is higher in uninfected respondents than in infected ones, indicating that the FCV has a greater impact on the mental health of uninfected than on infected individuals. This finding aligns with the results of Feng et al. (2020), which suggests that uninfected individuals have higher distress levels than infected ones. The uninfected individuals were more worried about the disease; they suspected every other person was infected with the virus; in some worsen conditions, uninfected people presumed that even the air in public places contained the virus. Thus, the FCV not only causes PD in infected but in uninfected ones.

## Theoretical Implications

This study contributes to the literature in several ways. Even though some of the most prominent research in consumer behavior has repeatedly demonstrated the influence of psychological factors on purchase behavior, but limited have explored their impact on GPB (Rathod and Bhatt, 2013; Adnan, 2014; Bashar and Saraswat, 2020). First, during the outbreak of COVID-19, customers were more concerned about their health and the environment; a shift from conventional to organic or green buying has been noticed, the factors that influenced this transition were unidentified. The study highlights the psychological factors that influence an individual’s behavior toward green products. Second, several studies have been conducted that used the duration of the COVID-19 pandemic (Koch et al., 2020; Alexa and Apetrei, 2021; Ali S. et al., 2021; Sun et al., 2021), but limited have done so with the FCV as an independent variable; this study contributes in this regard. Now, FCV is considered the most important predictor of green purchases. Third, the current study explores the mediating role of psychological variables, such as the MS and PD in mediating the relationship between FCV and GPB. However, these variables were infrequently used as mediators. Hence, the current study will contribute to the body of knowledge by exploring the rarely discussed direct and mediating relationship between FCV and GPB. Fourth, the findings align with the PMT; purchasing and consuming green products during the pandemic was a coping mechanism that reduced their stress, fear, and anxiety about death (Scarpa and Thiene, 2011; Al-Rasheed, 2020). In addition, green products are beneficial for their health and the environment. Last, the research on consumer behavior during the pandemic is still scarce due to its novelty; this study will also contribute in this regard.

## Practical Implications

The study’s findings have practical implications for marketers, manufacturers, retailers, and various other decision-makers. During the pandemic, as it was precisely related to the health of individuals, marketers should highlight the value and importance of green food products to consumers while acknowledging the possible negative consequences of conventional food products (Safuan et al., 2021). Since the consumer’s movement and accessibility to specific products were limited during the catastrophe, the marketers and retailers should use alternative channels to deliver healthy products to consumers. In Pakistan, the infected individuals were more motivated to make green purchases; manufacturers could consider them a potential market segment. Importantly, purchasing organic food discourages the superfluous use of fertilizer, which is good in production but damaging to the health and the environment. Moreover, strong collaboration among farmers, retailers, manufacturers, and consumers could be established and, as a result organic food production will be increased. Finally, this probably the first study to examine the effect of the FCV on green products purchasing behavior (i.e., organic food) which could potentially pave the way for a bright future for the green food market in Pakistan. As some short-term behaviors acquired to deal with the pandemic are more likely to become habit in the future, these findings can assist retailers and manufacturers in investing green products.

## Limitations and Future Research

This study has some limitations. First, the *R*^2^-value shows that GPB is 34.7% influenced by FCV, PD, and MS. Thus, there were other factors that influenced the GPB that must be considered while understanding the shift from conventional to green products. Second, out of 432 respondents, 172 were confirmed infected, and 260 were uninfected; the infected were more motivated in buying green products; future researchers should consider doing further research to figure out why uninfected individuals were less involved in GPB and should also include a large sample size. Third, according to PMT, green purchases are a coping mechanism. The findings are consistent with PMT; nevertheless, more research is needed to find out the other coping mechanism to alleviate the negative emotions during any catastrophe. Last, while this study is restricted to one realm, Pakistan, future research could be undertaken in a different context.

## Conclusion

This research aimed to identify the factors that influenced GPB during the pandemic and the relationship of these factors with GPB. After the data analysis, the results revealed that FCV, MS, and PD predict GPB. Among all aspects, FCV has the most potent predictive power in understanding GPB during the pandemic than MS and PD. Later, the mediation analysis added that the MS and PD mediate the effect of FCV on GPB. Hence, while the FCV is associated with adverse outcomes, it also leads to beneficial behaviors for every human being. In the MGA, the path FCV-GPB, FCV-PD, and FCV-MS significantly differ between infected and uninfected participants. Overall, the study’s findings corroborate the theory by confirming that FCV, PD, and MS were the threats that individuals faced during the pandemic, and that green purchases served as a coping strategy.

## Data Availability Statement

The original contributions presented in the study are included in the article/supplementary material, further inquiries can be directed to the corresponding author/s.

## Author Contributions

KS and SH: study conception and design. RH: data collection. KS, RH, and BM: analysis and interpretation of results. KS, RH, and SH: draft manuscript preparation. All authors reviewed the results and approved the final version of the manuscript.

## Conflict of Interest

The authors declare that the research was conducted in the absence of any commercial or financial relationships that could be construed as a potential conflict of interest.

## Publisher’s Note

All claims expressed in this article are solely those of the authors and do not necessarily represent those of their affiliated organizations, or those of the publisher, the editors and the reviewers. Any product that may be evaluated in this article, or claim that may be made by its manufacturer, is not guaranteed or endorsed by the publisher.
